# A Semilinear Parameter-Varying Observer Method for Fabric-Reinforced Soft Robots

**DOI:** 10.3389/frobt.2021.749591

**Published:** 2021-11-03

**Authors:** Phuc D.H. Bui, Joshua A. Schultz

**Affiliations:** Department of Mechanical Engineering, The University of Tulsa, Tulsa, OK, United States

**Keywords:** soft robotics, fabric-reinforced, inflatable, hysteresis, linear parameter-varying, observer design, contact force, sliding mode

## Abstract

This paper presents an observer architecture that can estimate a set of configuration space variables, their rates of change and contact forces of a fabric-reinforced inflatable soft robot. We discretized the continuum robot into a sequence of discs connected by inextensible threads; this allows great flexibility when describing the robot’s behavior. At first, the system dynamics is described by a linear parameter-varying (LPV) model that includes a set of subsystems, each of which corresponds to a particular range of chamber pressure. A real-world challenge we confront is that the physical robot prototype exhibits a hysteresis loop whose directions depend on whether the chamber is inflating or deflating. In this paper we transform the hysteresis model to a semilinear model to avoid backward-in-time definitions, making it suitable for observer and controller design. The final model describing the soft robot, including the discretized continuum and hysteresis behavior, is called the semilinear parameter-varying (SPV) model. The semilinear parameter-varying observer architecture includes a set of sub-observers corresponding to the subsystems for each chamber pressure range in the SPV model. The proposed observer is evaluated through simulations and experiments. Simulation results show that the observer can estimate the configuration space variables and their rate of change with no steady-state error. In addition, experimental results display fast convergence of generalized contact force estimates and good tracking of the robot’s configuration relative to ground-truth motion capture data.

## 1 Introduction

Soft robots are designed to safely interact with and adapt to their surrounding environment (Kim et al., 2013). Their bodies are soft and deformable so that they can be compliant in narrow spaces or when they make contact with objects (Bauer et al., 2014; Rus and Tolley, 2015). Hence, body deformation and contact forces are the main issues that a soft robot has to confront during operation. In order to complete a task, the controller of a soft robot needs to know the current states of the robot as well as the information about disturbances such as contact forces acting on the robot body. However, due to the continuously deformable nature of a soft robot, perceiving such information is still technically challenging (Santina et al., 2020). Despite recent advances in soft sensor technologies, it’s almost impossible to accurately measure those quantities by sensors integrated into the robot body. A good alternative is using an observer for state and generalized contact force estimation. There have been several authors working on the design of observers for soft robots. Santina et al. (2020) designed an observer combined with machine learning to detect contacts on a soft robot. The work focuses on characterizing contact events on the soft robot when only knowledge of its posture and actuation are available. Zhang et al. (2016) used finite elements to estimate the configuration matrices. The observer was based on the real-time finite element method (FEM) that combined feedback signals from the real robot and model information from the robot simulated by FEM. Gillespie et al. (2016) used a Kalman filter to integrate accelerations and angular velocities for robot postures. Ataka et al. (2016) used a multi-stage Extended Kalman Filter to estimate the soft robot poses. Rone and Ben-Tzvi (2013) estimated the robot states using the displacement of passive cables. Srivatsan et al. (2014) used Lie algebra to estimate the shape of a medical snake robot. In general, due to the complication of the soft robots, a lot of efforts were made in the aforementioned works to estimate some states or to detect a contact event. However, there has been no model-based observer designed to estimate both soft robot state and contact forces. It is worth noting that designing observers for soft robots is a challenging task because they are highly nonlinear systems and modeling processes for them are complicated. Currently there are three predominant approaches to model a soft robot: Piece-wise Constant Curvature (PCC) (Webster and Jones, 2010), discrete Cosserat models (Renda et al., 2018) and 3D Finite Element Models (Connolly et al., 2015). The PCC model seems to be the simplest one among the three modeling approaches due to its simplifications and assumptions but even so, a model-based observer for a PCC soft robot is still very difficult to obtain.

In this paper, we introduce a new observer design for our recently developed fabric-reinforced inflatable soft robot “Squishy” Williamson et al. (2021). It is a model-based observer built around the new disc-thread model state parameterization Schultz et al. (2020). We use a third-order sliding mode approach, which is powerful and has high robustness, to design the observer so that it can estimate a large number of robot states and generalized contact forces. The state estimation uses position data from markers placed at several locations on the robot body. The estimator computes an estimated contact force by considering it to be a disturbance to the disc-thread model prediction. We will compare this to contact force measurements acquired experimentally using a force sensor. The advantage of the proposed observer over the existing ones is that it can estimate a large number of both the robot states and generalized contact forces. A preliminary version of this observer architecture appears in the proceedings of ICRA 2021 (Bui and Schultz, 2021), but this article includes additional details in the formulation of the observer, new runs of the simulation with a refinement to the model, and the experimental validation that did not appear in the conference paper. In addition, the discussion of the hysteresis behavior is new to this work. For the modeling part, this paper uses the recently developed disc-thread approach to model a fabric-reinforced inflatable soft robot. In the disc-thread modeling approach, the soft robot is discretized into *N* discs connected by *N* − 1 threads to represent a kinematic chain of rigid bodies, and the equations of motion are formulated using the Langrangian method. Since the modeling process produces ODEs in the manner of a traditional robot, the observer is model-based and has a closed form for the same class of robot models. In order to design a model-based observer, at first we developed a linear parameter-varying (LPV) system which is comprised of a set of linear systems, each corresponding to different working pressures, to represent the whole nonlinear soft robot. This LPV model is also suitable to design a model-based controller in the future.

This paper also discusses the hysteresis behavior of the soft robot. Like almost every other viscoelastic system, this silicone soft robot follows a different path when inflating than it does when deflating. This behavior is interesting but it increases the complexity in the modeling and control process of the robot because the controller must keep track of whether the robot is inflating or deflating. There have been only a few papers working on the hysteresis of soft robots such as the work of Hošovský et al. (2016) where hysteretic behavior of a two-DOF soft robotic arm is analyzed, or the work of Vo-Minh et al. (2011) where the hysteresis is modeled using several Maxwell-slip elements each with different stiffness and saturation force. Our paper applies a modified generalized Duhem model to describe the hysteresis behavior of the robot. The advantage of this approach is that it avoids backward-in-time definitions, making it suitable for observer and controller design. We call the final system the semilinear parameter-varying (SPV) model, which is the proposed LPV system that accounts for the hysteresis behavior.

This article is structured as follows. In Section 2.1, we introduce our soft robot and review the disc-thread model approach as well as the formulation of the equations of motion. The LPV system is addressed in Section 2.2. Section 2.3 discusses the hysteresis loop behavior and the SPV model of the soft robot. Section 2.4 describes the observer design for the soft robot. The settings of simulations and experiments are described in Section 2.5. Section 3 shows the simulation and experimental results of the observer. The conclusion is presented in Section 4.

## 2 Materials and Methods

### 2.1 The Fabric-Reinforced Inflatable Soft Robot and Disc-Thread Model

**FIGURE 1:**
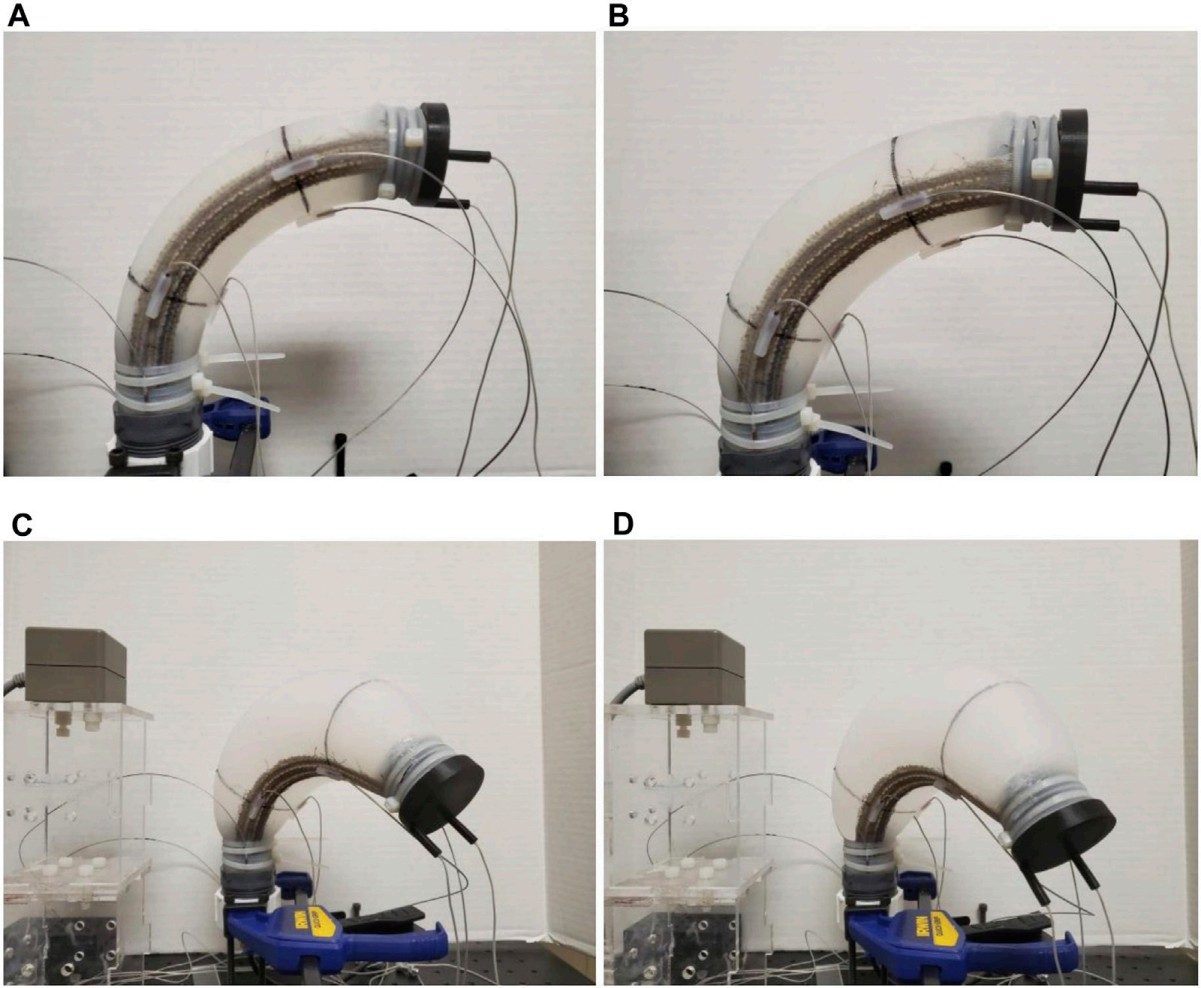
The poses of the fabric-reinforced inflatable soft robot at four different pressures, **(A)** At 1.5 psi, **(B)** at 2.5 psi, **(C)** at 3 psi, **(D)** at 3.5 psi [Reprinted from (Bui and Schultz, 2021) with permissions].

Our soft robot, “Squishy”, is an inflatable elastomeric chamber made of Smooth-On Dragon Skin 30 silicone that has a thin band of fabric embedded longitudinally to reinforce one side of the robot. The details of the manufacturing process and basic characteristics, such as the workspace volume and inflation-displacement behavior can be found in (Williamson et al., 2021). The undeformed shape of the chamber and the fabric band are 2D circular arcs. The chamber is closed by two caps at either end. One of the two caps has an inlet so that compressed air can be pumped in. When inflated pneumatically, the unreinforced side can undergo large strains while the fabric side maintains a constant length. This causes the chamber to bend and twist. Because of the fabric’s placement on the robot, its tip can trace 3D curves rather than just 2D curves as would a straight robot. Figure 1 shows the poses of the robot at some different working pressures.

The higher the robot chamber pressure is, the more it expands and the more it bends down and twists. The entire length of the robot arm could be used to perform some tasks such as scraping or pushing against objects in its environment. The advantage of this soft robot is that it can perform some tasks in 3D space with only one actuator (which is the controlled compressed air **P**). It should not damage objects, nor itself in the case of collisions due to the soft body. When the robot is not in use, it can be deflated, rolled, and stored in a tiny box to save space. The robot can also be included as a single module within a larger system to perform more sophisticated tasks.

**FIGURE 2:**
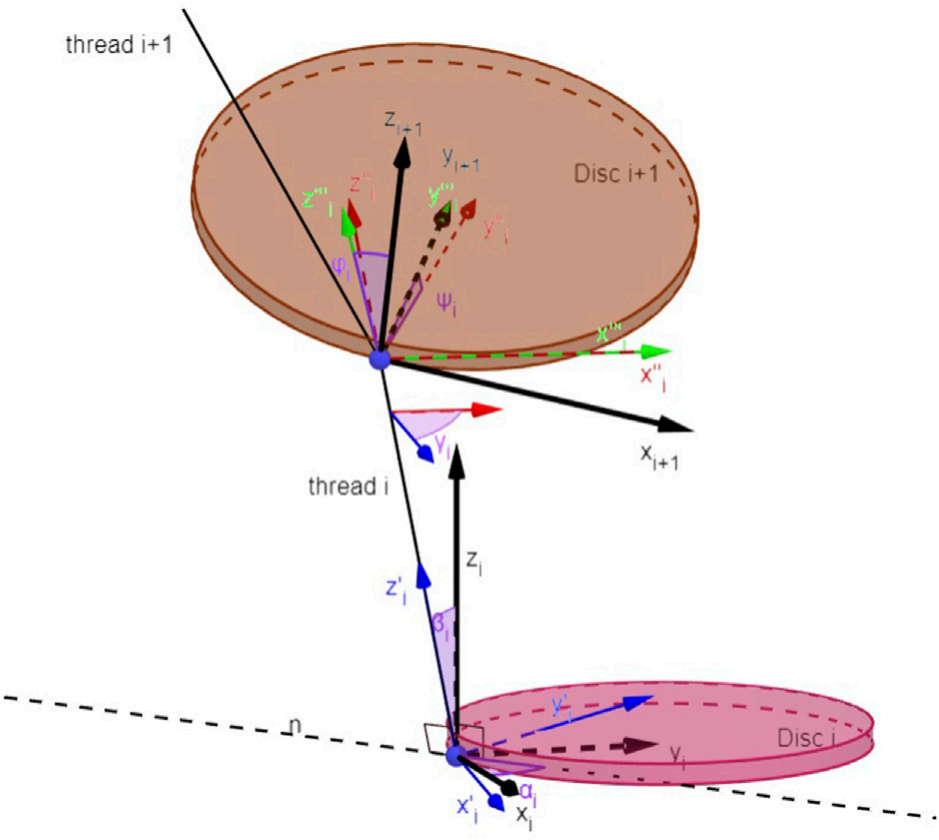
Disc-thread model [Reprinted from (Bui and Schultz, 2021) with permissions].

To represent the motion of the robot, which is a continuum, using a finite set of variables, we use the Disc-thread model, which is briefly reviewed in this section. This was introduced in Schultz et al. (2020), and has the advantages of describing the bending and twisting of the soft robot in an easily customized fashion by a set of differential equations. In this approach, we discretize the chamber longitudinally into a sequence of *N* discs, each connected to its neighbors on either side by a single inextensible thread (representing the fabric reinforcement). Each disc is considered to be a rigid body and is constrained only by the thread. The frame assignments and their implications are illustrated in Figure 2. Each pose of the soft robot is derived from the relative position and orientation between pairs of adjacent discs. Each disc is parameterized by five generalized coordinates. The thread *i* is parameterized by two angles *α*_*i*_, *β*_*i*_ ∈ [0; *π*]. The subsequent frames will involve a translation along the inextensible thread (of a fixed distance *ℓ*_*i*_), together with three other rotations *γ*_*i*_, *ψ*_*i*_ and *ϕ*_*i*_, where the twisting of the robot is accounted by *γ*_*i*_. So by relying on these fictitious discs and threads as well as angles from *α*_1_ to *ϕ*_5(*N*−1)_, this approach models the soft robot as a kinematic chain of rigid bodies. However, because the joints are not prismatic or revolute this model contains a higher number of variables than a traditional kinematic chain.

With the kinematics of the soft robot fully defined by the disc-thread model, we formulate the Lagrangian and took its derivatives to find the equations of motion using the Euler-Lagrange formulation. The elasticity of the air is modeled by treating the air as an ideal gas, which exerts a normal force on the *N*th disc in the *z*_*N*_ direction. The elasticity of the walls is modeled by connecting springs from some origin on disk *i* to insertion on disk *i* + 1. The equations of motion has the familiar form:

$$\frac{d}{dt}\left(\frac{\partial L}{\partial \dot{q}}\right)-\frac{\partial L}{\partial q}+A_{q}^{T}\lambda =Q$$ (1)

where $q\in R^{5\left(N-1\right)}$ is the configuration vector containing *α*_1_⋯*ϕ*_*N*−1_ variables, $\frac{\partial L}{\partial q}$ is the partial derivative of the Lagrangian with respect to each generalized variable, $Q\in R^{5\left(N-1\right)}$ is the generalized forces due to the internal pressure acting on the surface of the last disc, and $A_{q}\in R^{5\left(N-1\right)\times 5\left(N-1\right)}$ is the Jacobian of the Pfaffian constraints arranged so that it can be multiplied by the vector of Lagrange multipliers $\lambda \in R^{5\left(N-1\right)}$ . In this work, we are trying to observe the robot configuration (and contact force, if applicable), whether the robot is in free space or in contact. In contact the product $A_{q}^{T}\lambda$ will be nonzero and we replace it by **F**_*c*_, where “*c*” denotes the effects of the contact force on the robot’s configuration space. This term will have the same units as the generalized force **Q**. By using the canonical momenta vector $p=\frac{\partial L}{\partial \dot{q}}=M\dot{q}$ with the mass matrix $M\in R^{5\left(N-1\right)\times 5\left(N-1\right)}$ , we can rewrite the above equation of motion in the state space form as:

$$\left(\begin{array}{c} \dot{q}\\ \dot{p} \end{array}\right)=\left(\begin{array}{cc} 0 & M^{-1}\\ 0 & 0 \end{array}\right)\left(\begin{array}{c} q\\ p \end{array}\right)+\left(\begin{array}{c} 0\\ \frac{\partial L}{\partial q}+Q-F_{c} \end{array}\right)$$ (2)

### 2.2 Linear Parameter-Varying Modeling of the Soft Robot

The inflatable soft robot described in Section 2.1 will operate at a range of pressures. Because the analytical expressions of **M**, ∂L/∂q and **Q** are functions of generalized coordinate **q** and the input pressure **P**, the equations of motion of the soft robot are not constant but change corresponding to each pressure (operating point). Note that the expressions of these matrices are complicated because they include partial derivatives of expressions of many variables and they are varying nonlinearly with pressure. Therefore, if we evaluate **M**, ∂L/∂q and **Q** at a particular pressure and obtain the numerical values at that operating point, we can retrieve an affine linear-time-invariant state space that represents the soft robot nearby that operating point. In order to describe the robot throughout its entire range of motion, we divide the entire system into *K* subsystems fixed to a sequence of operating points, by evaluating the constituent matrices at corresponding pressures. This action forms a switched LPV (linear parameter-varying) system. Note that input pressure is chosen as the switching condition because it is the only input that changes the robot configuration. It is also measured easily and we don’t have to know the robot pose explicitly to select the correct operating points. The LPV system consists of a set of linear systems whose state-space operational modes are driven by an underlying decision process based on the current input pressure. The LPV system is expressed as the linear state space system:

$$\begin{array}{c} \dot{\bar{x}}\left(t\right)=A_{v\left(u\right)}\bar{x}\left(t\right)+B_{v\left(u\right)}u\left(t\right)+\Theta_{v\left(u\right)}\\ \bar{y}\left(t\right)=C\bar{x}\left(t\right) \end{array}$$ (3)

where $\bar{x}={\left[q\;\;p\right]}^{T}={\left[x_{1}\;\;x_{2}\right]}^{T}$ , the output $y\in R^{m}$ consists of position measurements from motion capture markers attached to the body of the robot. The output is related to the states by $C=\left[C_{1}^{m\times m}\;0\right]\in R^{m\times n}$ which results from the linearization of the nonlinear functions mapping the measured data to the world frame. The switching rule *v*(*u*) ∈ *S* ^*K*^ *≐*{1, 2, …*K*} depends on input pressure **P**. For each *j* ∈ *S* ^*K*^, the subsystem matrices *A*_*j*_, *B*_*j*_ and Θ_*j*_ are evaluated at the **q** corresponding the pose at the pressure **P**_*j*_, resulting in constant matrices and have the forms:

$$\begin{array}{c} A_{j}=\left[\begin{array}{cc} 0 & M_{j}^{-1}\\ 0 & 0 \end{array}\right]\\ B_{j}=\left[\begin{array}{c} 0\\ Q_{j} \end{array}\right]\\ \begin{array}{c} \Theta_{j} \end{array}=\left[\begin{array}{c} 0\\ {\left(\frac{\partial L}{\partial q}\right)}_{j}-F_{cj} \end{array}\right] \end{array}$$ (4)

The number of operating points and their distribution are carefully selected so that the switched LPV system can adequately represent the whole continuous system. The system is considered under the following Assumption.

Assumption 1 The generalized contact forces in Eq. 4 satisfy the following conditions: **F**_*cj*_ has a derivative and both are upper bounded as ‖**F**_*cj*_‖ ≤ *L*_*j*_, $\|{\dot{F}}_{cj}\|\leq L_{j}$ .

### 2.3 Hysteresis Loop and Semilinear Parameter-Varying Model of the Soft Robot

**FIGURE 3:**
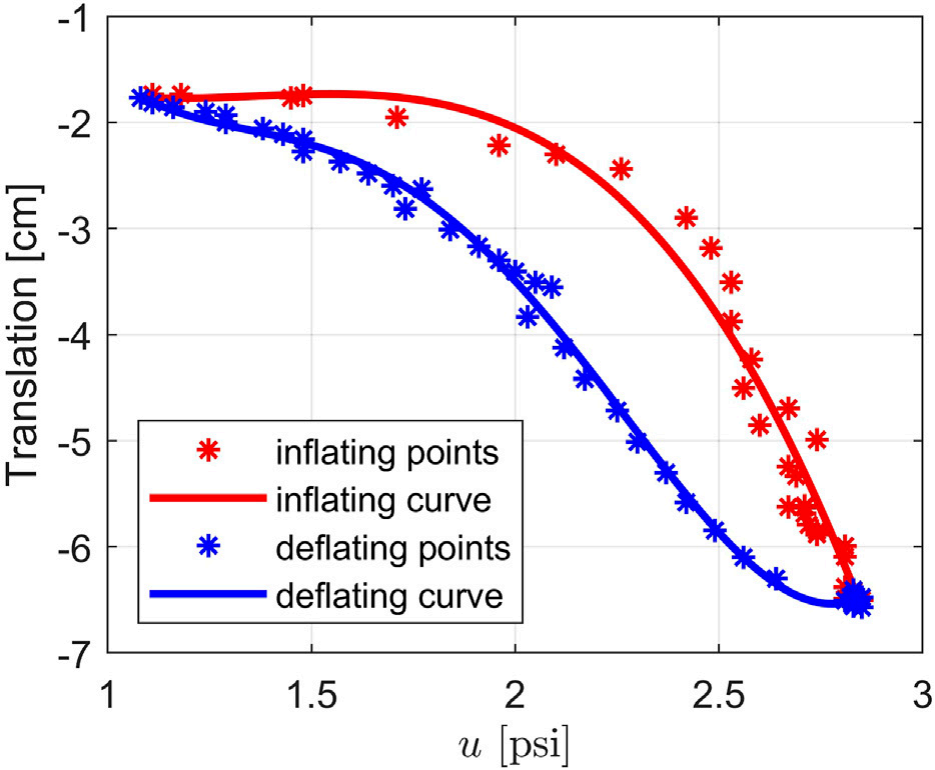
The hysteresis loop describing the translation of the tip of the soft robot in *Y* direction.

Before continuing with the discussion of the observer we discuss the hysteresis behavior of the soft robot. Hysteresis is defined as a “special type of memory-based relation between the input and the output” (Macki et al., 1993) or “rate-independent memory effect” (Visintin, 1994). Although there have been variations of the definition, hysteresis is generally used to describe a dynamical system with a phase lag that depends on the input. Hysteresis systems can be found in different areas such as physics, chemistry, engineering or economics. For our robot, we conducted experiments and observed that since our robot is made of silicone and is stretchable, its behavior is described as a hysteresis loop where the body of the robot follows different curves depending on the changing direction of the input pressure. Specifically, by using the motion tracking system to inspect the tip position in the *Y* direction of the robot, we could see that the tip followed the red curve while the chamber was inflating and followed the blue curve while the chamber was deflating, as shown in Figure 3. Note that the curves are generated by polynomial regressions using data (the discrete points) collected from the motion tracking system. It can be seen that the deflating curve has a higher slope than that of the inflating curve. In other words, when deflating, the robot can reach a desired position faster than when it is inflating. This behavior of the inflatable robot is interesting but it increases the complexity in the system observation and control design.

Since the system configuration depends on the direction of the input pressure, from the idea of an LPV system in Eq. 3, we can express it as a generalized Duhem model as follows (Visintin, 1994):

$$\begin{array}{c} \dot{\bar{x}}\left(t\right)=\left(A_{v\left(u\right)}x\left(t\right)+B_{v\left(u\right)}u\left(t\right)+\Theta_{v\left(u\right)}\right)g\left(\dot{u}\left(t\right)\right)\\ \bar{y}\left(t\right)=C\bar{x}\left(t\right) \end{array}$$ (5)

where

$$\begin{array}{c} g\left(w\right)=\left\{\begin{array}{c} \begin{array}{c} h^{+}w\;\text{if}\;w\geq 0\\ h^{-}w\;\text{if}\;w<0 \end{array}\; \end{array}\right. \end{array}$$ (6)

where *h* ^+^ and *h* ^−^ are different functions corresponding to the direction of the input signal.

More specifically, this hysteretic system can be writen as an SPV system as:

$$\begin{array}{c} \dot{\bar{x}}\left(t\right)≜\dot{x}\left(u\right)=\left\{\begin{array}{c} \begin{array}{c} A_{v\left(u\right)}^{+}x\left(t\right)+B_{v\left(u\right)}^{+}u\left(t\right)+\Theta_{v\left(u\right)}^{+}\;\text{if}\;\dot{u}\geq 0\\ A_{v\left(u\right)}^{-}x\left(t\right)+B_{v\left(u\right)}^{-}u\left(t\right)+\Theta_{v\left(u\right)}^{-}\;\text{if}\;\dot{u}<0 \end{array}\; \end{array}\right.\\ \bar{y}\left(t\right)≜y\left(u\right)=C\bar{x}\left(t\right) \end{array}$$ (7)

This SPV system contains twice the number of subsystems in Eq. 3, including a half subsystem to describe the LPV system while the robot is inflating and the other half to represent the system in the deflating direction of the input. The switching action takes place when the control input changes its direction.

In the current formulation, we have to do backward-in-time identification for the hysteresis curve and the input pressure, which are not suitable when designing conventional observers and controllers. To avoid such kind of identification and checking the derivative of the input, we applied a transformation by introducing a monotonically increasing independent variable, $\bar{u}\in \left[u_{min},2u_{max}-u_{min}\right]$ . The transformation is inspired by the work of Oh and Bernstein (2005). This helps us to rewrite the model as a new semilinear Duhem model as follows:

$$\begin{array}{c} \dot{x}\left(\bar{u}\right)=\left\{\begin{array}{c} \begin{array}{c} A_{v\left(\bar{u}\right)}^{+}x\left(\bar{u}\right)+B_{v\left(\bar{u}\right)}^{+}\bar{u}+\Theta_{v\left(\bar{u}\right)}^{+}\;\text{if}\;u_{min}\leq \bar{u}\leq u_{max}\\ A_{v\left(\bar{u}\right)}^{-}x\left(\bar{u}\right)+B_{v\left(\bar{u}\right)}^{-}\bar{u}+\Theta_{v\left(\bar{u}\right)}^{-}\;\text{if}\;u_{max}<\bar{u}\leq 2u_{max}-u_{min} \end{array}\; \end{array}\right.\\ y\left(\bar{u}\right)=Cx\left(\bar{u}\right) \end{array}$$ (8)

where

$$\bar{y}\left(\bar{u}\right)≜\left\{\begin{array}{c} \begin{array}{c} {\bar{y}}^{+}\left(\bar{u}\right)\;\text{if}\;u_{min}\leq \bar{u}\leq u_{max}\\ {\bar{y}}^{-}\left(u_{max}+u_{min}-\bar{u}\right)\;\text{if}\;u_{max}\leq \bar{u}<2u_{max}-u_{min} \end{array}\; \end{array}\right.$$ (9)

**FIGURE 4:**
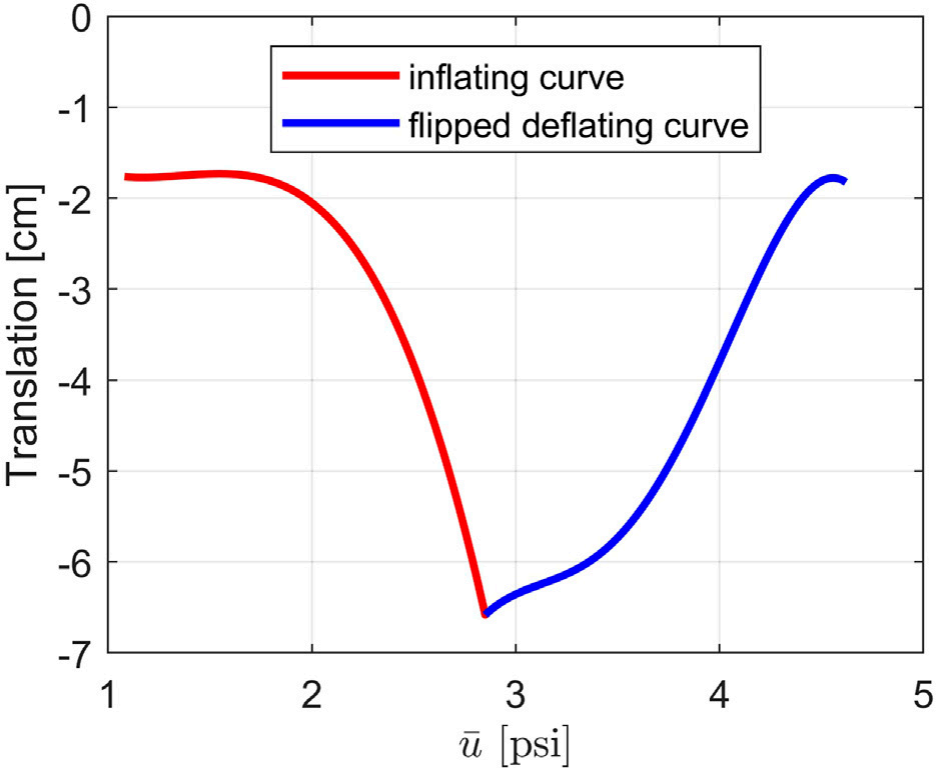
The new hysteresis curves describing the translation of the tip of the soft robot in *Y* direction with respect to $\bar{u}$

The new hysteresis curves describing the tip of the robot are displayed in Figure 4 where the inflation curve is the red line and the deflation curve is the blue line. Compared to the version in Figure 3, the deflating curve is now flipped over so that the two curves form a continuous function with respect to the new monotonically increasing input $\bar{u}$ . A system identification process has been performed to define each matrix in the SPV system in Eq. 8 at each working point to complete all of its subsystems. Then the SPV model can be used for observer and controller design.

### 2.4 Observer Design for the Soft Robot

Due to the deformable body of the soft robot, the numerical values of **q**, **p** and **F**_*c*_ in Eq. 2 can not be obtained directly from measurements from the sensors we are using. The model of the soft robot in this study includes a large number of state variables, and we need an observer to estimate both states and contact forces. For these reasons, we selected the high-order sliding mode observer, because it has a simple rule to select the observer gains, no matter the size of the state space in observation; and based on our knowledge, it is the best observer that works well for both states and disturbance observation. The other alternatives are more challenging to be applied to our system. The following Lemma is re-stated to summarize the result of finite-time stability of the dynamical system in the study of Levant and Livne (Levant, 2003), which is applied to develop the observer:

Lemma 1 Consider the following system:

$$\begin{array}{cc} {\dot{\varepsilon }}_{0} & =-\lambda_{0}|\varepsilon_{0}{|}^{n/n+1}sign\left(\varepsilon_{0}\right)-\eta_{0}\varepsilon_{0}+\varepsilon_{1},\\ {\dot{\varepsilon }}_{1} & =-\lambda_{1}|\varepsilon_{1}-{\dot{\varepsilon }}_{0}{|}^{\left(n-1\right)/n}sign\left(\varepsilon_{1}-{\dot{\varepsilon }}_{0}\right)-\eta_{1}\left(\varepsilon_{1}-{\dot{\varepsilon }}_{0}\right)\\ & \;+\varepsilon_{2},\\ & \;\ldots \\ {\dot{\varepsilon }}_{n-1} & =-\lambda_{n-1}|\varepsilon_{n-1}-{\dot{\varepsilon }}_{n-2}{|}^{1/2}sign\left(\varepsilon_{n-1}-{\dot{\varepsilon }}_{n-2}\right)\\ & \;-\eta_{n-1}\left(\varepsilon_{n-1}-{\dot{\varepsilon }}_{n-2}\right)+\varepsilon_{n},\\ {\dot{\varepsilon }}_{n} & =-\lambda_{n}sign\left(\varepsilon_{n}-{\dot{\varepsilon }}_{n-1}\right)-\eta_{n}\left(\varepsilon_{n}-{\dot{\varepsilon }}_{n-1}\right)-\frac{1}{L_{0}}f\left(t\right) \end{array}$$ (10)

*where* *n* *is the relative degree,* *ɛ*_0_, …, *ɛ*_*n*_ *are the state variables,* *λ*_0_, …, *λ*_*n*_ *and* *η*_0_, …, *η*_*n*_ *are appropriate positive scalar constants and the perturbation* *f*(*t*) *satisfies the condition* |*f*(*t*)| ≤ *L*_0_ *with* *L*_0_ *a proper positive constant. Then the system converges to the origin in finite time.*

Given position data from the marker set, we built a set of observers based on the third order sliding mode approach of Levant (2003), which has fast convergence and high robustness. The number of observers is the same as the number of subsystems in Eq. 8 The observer for subsystem *j* is of the form:

$$\begin{array}{cc} {\dot{\hat{x}}}_{1j} & =M_{j}^{-1}{\hat{x}}_{2j}-k_{1j}L_{j}^{1/3}\|{\hat{x}}_{1j}-x_{1j}{\|}^{2/3}sign\left({\hat{x}}_{1j}-x_{1j}\right)\\ & \;-k_{2j}\left({\hat{x}}_{1j}-x_{1j}\right)\\ {\dot{\hat{x}}}_{2j} & =-k_{3j}L_{j}^{1/2}\|M_{j}^{-1}{\hat{x}}_{2j}-{\dot{\hat{x}}}_{1j}{\|}^{1/2}sign\left(M_{j}^{-1}{\hat{x}}_{2j}-{\dot{\hat{x}}}_{1j}\right)\\ & \;-k_{4j}\left(M_{j}^{-1}{\hat{x}}_{2j}-{\dot{\hat{x}}}_{1j}\right)+{\left(\frac{\partial L}{\partial q}\right)}_{j}+Q_{j}+{\hat{F}}_{cj}\\ {\dot{\hat{F}}}_{cj} & =-k_{5j}L_{j}sign\left(M_{j}^{-1}{\hat{x}}_{2j}-{\dot{\hat{x}}}_{1j}\right)\\ & \;-k_{6j}L_{j}\left(M_{j}^{-1}{\hat{x}}_{2j}-{\dot{\hat{x}}}_{1j}\right) \end{array}$$ (11)

where ${\hat{x}}_{1j}$ , ${\hat{x}}_{2j}$ are vectors of estimated states, *k*_1*j*_ to *k*_6*j*_ are the observer gains to be designed and ${\hat{F}}_{cj}$ is the vector of estimated generalized contact forces. Note that **x**_1*j*_ can be obtained from the multiplication of the inverse of *C*_1_ and **y**. Since we have more outputs than the number of states (one marker provides three outputs including the 3D coordinate of that marker in *X*, *Y*, *Z* direction), we can form a non-singular square matrix *C*_1_ so that it is invertible.

**Theorem** 1 For system Eq. 8, if the observer set is designed as in Eq. 11 and the observer gains are selected properly, then the estimated states and contact forces will converge to the true values in finite time, which follows from Lemma 1 (Levant, 2003).

Proof. The estimation error variables are defined by the following formulation:

$$\begin{array}{cc} \epsilon_{1j} & =\frac{{\hat{x}}_{1j}-x_{1j}}{L_{j}}\\ \epsilon_{2j} & =\frac{M_{j}^{-1}\left({\hat{x}}_{2j}-x_{2}\right)}{L_{j}}\\ & =\frac{M_{j}^{-1}{\hat{x}}_{2j}-{\dot{\hat{x}}}_{1j}}{L_{j}}\\ & =\frac{M_{j}^{-1}\left({\hat{x}}_{2j}-M{\dot{\hat{x}}}_{1j}\right)}{L_{j}}\\ \epsilon_{3j} & =\frac{{\hat{F}}_{cj}-F_{cj}}{L_{j}} \end{array}$$ (12)

the dynamics of the estimation errors are then obtained as:

$$\begin{array}{cc} {\dot{\epsilon }}_{1j} & =\frac{1}{L_{j}}\left(-k_{1j}L_{j}^{1/3}\|L_{j}\epsilon_{1j}{\|}^{2/3}sign\left(\epsilon_{1j}\right)-k_{2j}\left(L_{j}\epsilon_{1j}\right)\right.\\ & \;+\left.M_{j}^{-1}{\hat{x}}_{2j}-M_{j}^{-1}x_{2}\right)\\ & =-k_{1j}\|\epsilon_{1j}{\|}^{2/3}sign\left(\epsilon_{1j}\right)-k_{2j}\left(\epsilon_{1j}\right)+\epsilon_{2j},\\ {\dot{\epsilon }}_{2j} & =\frac{1}{L_{j}}\left(-k_{3j}L_{j}^{1/2}\|L_{j}\epsilon_{2j}{\|}^{1/2}sign\left(\epsilon_{2j}\right)-k_{4j}\left(L_{j}\epsilon_{2j}\right)\right.\\ & \;+\left.{\hat{F}}_{cj}-F_{cj}\right),\\ & =-k_{3j}\|\epsilon_{2j}{\|}^{1/2}sign\left(\epsilon_{2j}\right)-k_{4j}\left(\epsilon_{2j}\right)+\epsilon_{3j}\\ {\dot{\epsilon }}_{3j} & =-k_{5j}sign\left(\epsilon_{2j}\right)-k_{6j}\epsilon_{2j}-\frac{1}{L_{j}}{\dot{F}}_{cj} \end{array}$$ (13)

If the conditions in Assumption 1 are satisfied, it follows from Lemma 1 that system Eq. 13 is finite-time stable, which implies that the estimation variables converge to true variables in finite time. According to the rule recommended by Levant (2005), the convergence can be guaranteed by defining the gains as *k*_1*j*_ ≥ 3, *k*_3*j*_ ≥ 1.5, *k*_5*j*_ ≥ 1.1 for this third-order system. Note that the larger the gains, the faster the convergence and the higher sensitivity to input noises and the sampling step. *k*_2*j*_, *k*_4*j*_, *k*_6*j*_ can be selected through trials in the simulation.

**FIGURE 5:**
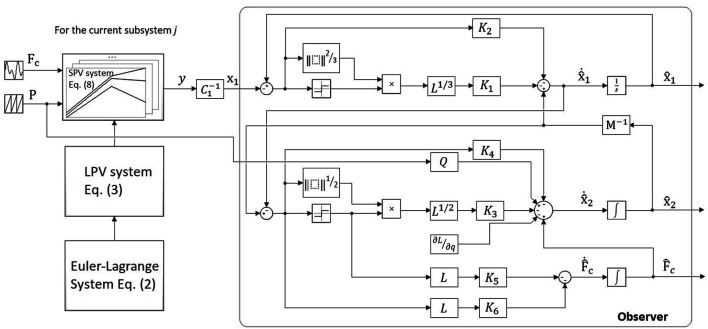
Block diagram of the observer.

The block diagram in Figure 5 summarizes the design process for the robot systems and the observer. The observer block stands for the sub-observer *j* serving the corresponding subsystem *j* working at a certain pressure value.

### 2.5 Simulation and Experimental Validation

**FIGURE 6:**
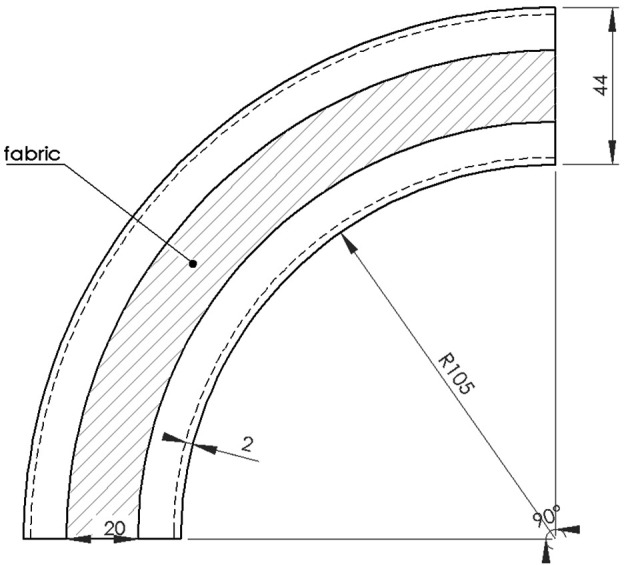
Soft robot dimension.

**FIGURE 7:**
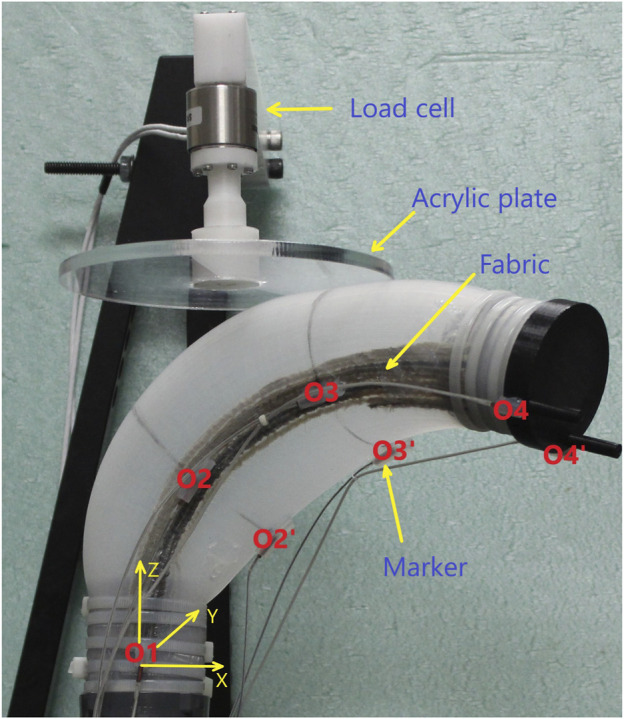
The contact case at disc three for contact forces measurement and 7 markers for the robot poses tracking.

**FIGURE 8:**
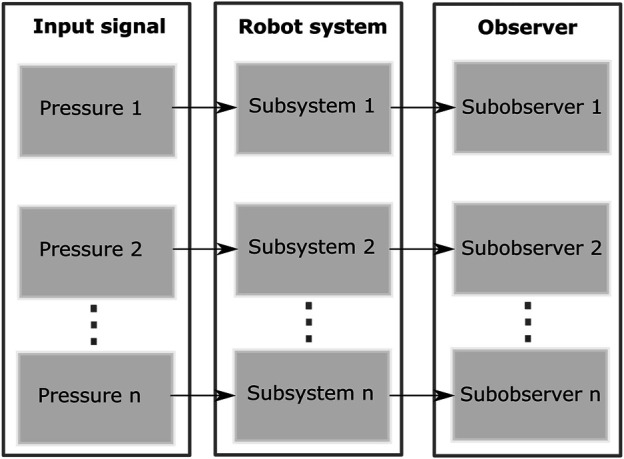
Switching rule.

The soft robot and position of the fabric can be seen in Figure 1 with the detailed dimensions (in mm) of the undeformed shape are given in Figure 6. To apply the Disc-Thread modeling into the simulations and experiments, our soft robot is discretized into four discs and three threads. The first and last discs are at the base and the tip of the robot, correspondingly. The second disc is considered to go through two points *O*_2_ and $O_{2}^{\prime }$ and the third disc is consider to go through *O*_3_ and $O_{3}^{\prime }$ (see Figure 7). The thread lengths are set at *ℓ*_1_ = 7 cm, *ℓ*_2_ = 9 cm and *ℓ*_3_ = 7 cm. Note that the robot can be modeled with a higher number of discs but it will result in longer expressions without much improvement in model accuracy. For a robot of this size and aspect ratio, this four-disc model can adequately describe its behavior. We selected four operating points at different increasing pressures at (1.5, 2.5, 3, 3.5) psi (see Figure 1) and evaluate **M**, ∂L/∂q and **Q** at these points to generate four corresponding subsystems that represent the entire working range of the soft robot during its inflation. The set of these matrices are provided in the supplemental data. Note that these four subsystems are the same for the LPV system and the inflating branch of the SPV system. Considering that working with the whole SPV system is too long for this paper, and it does not improve the illustration of the observer performance, we only use the inflating branch of the SPV system for our simulation and experiment. A set of sub-observers is designed using the form of Eq. 11. The observer gains are chosen as *k*_1_ = 3, *k*_2_ = 4, *k*_3_ = 1.5, *k*_4_ = 3, *k*_5_ = 1.1, *k*_6_ = 2 for all sub-observers. The disturbance is assumed to be bounded by *L* = 20. At the run-time of the robot, when a new subsystem is switched on due to the changing pressure, the new corresponding sub-observer with updated system matrices is switched on as well. The switching rule is illustrated in Figure 8.

The proposed observer was validated by simulations and experiments. The simulations were performed in Matlab Simulink using S-functions for the dynamics of the plant and observer. The experiments were conducted in our laboratory environment. They are described in detail as follows:

Simulation 1: We examined the ability of the observer to estimate the space variables *q*_*i*_ at four operating points during quasistatic motion in free space for the whole continuous system.
Simulation 2: We assumed that the soft robot is undergoing a motion in free space going through all four operating points with a constant angular acceleration to check the estimation of the rates of changes of each *q*_*i*_.

**FIGURE 9:**
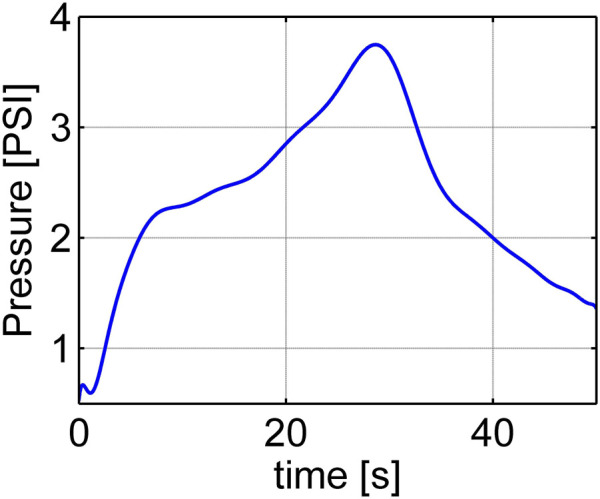
Input pressure for the experiment to estimate contact forces and the robot poses.

Experiment 1: We conducted this experiment to gather data from a real contact event between the soft robot and its surrounding environment. The data will be used to validate the observer performance after it estimates the generalized contact force. In order to measure the real contact forces, we built a system that includes a load cell connected to an acrylic plate on one end and connected to a frame on the other (see Figure 7). The load cell is an Interface 6A27 which can measure forces simultaneously in three mutually perpendicular axes and three simultaneous torques about those same axes. Initially, the robot was kept in a stable position and the plate was located at 3.38 cm above the robot. We inflated and deflated the robot with the input pressure history shown in Figure 9. We observed that at first the robot raised its head and then bent down and twisted (as can be seen in Figure 1) when inflated, and returned to its original pose when deflated. The input pressure and the initial gap between the plate and the robot were chosen so that the robot was in contact with the plate surface at the location corresponding to disc three when the chamber pressure was in the range [1.5–3.3] psi. During the contact event, the contact forces in each direction were recorded by the load cell. Then a set of 15 generalized contact forces (which have units of torque) were calculated using $F_{c}=J_{bc}^{T}F$ (where *F* is the three components of the contact forces measured by the load cell and *J*_*bc*_ is the relative Jacobian between the base and the contact point). These values were the ground-truth generalized contact forces. To simulate the observer estimating generalized contact forces, the measured forces were considered as disturbances to the robot model. When we ran the Simulink model of the plant and the observer, the observer could detect the disturbances (generalized contact forces) and produced the close estimates of these same measured forces.

Experiment 2: In this experiment, the robot was moving in free space without any contact with the environment. We inflated and deflated the robot with the same input pressure history shown in Figure 9. Since the disc-thread model represents a discretization of a continuum soft robot, the discs themselves are fictitious and do not exist in the physical system. Thus, there is no ground-truth value against which to directly compare the state estimates (*α*_1_ to *ϕ*_3_). However, we can compute what the marker locations *should* be based on the estimated states and compare them to the measured marker locations. The poses assumed by the robot during its movement were recorded by seven markers of the Polhemus electromagnetic motion tracking system. The 3D location of the markers on the body of the robot are considered to be the measured output of this robot system. We then feed the data measured from the markers into the observer into the Simulink model to estimate the soft robot poses.

## 3 Results

**FIGURE 10:**
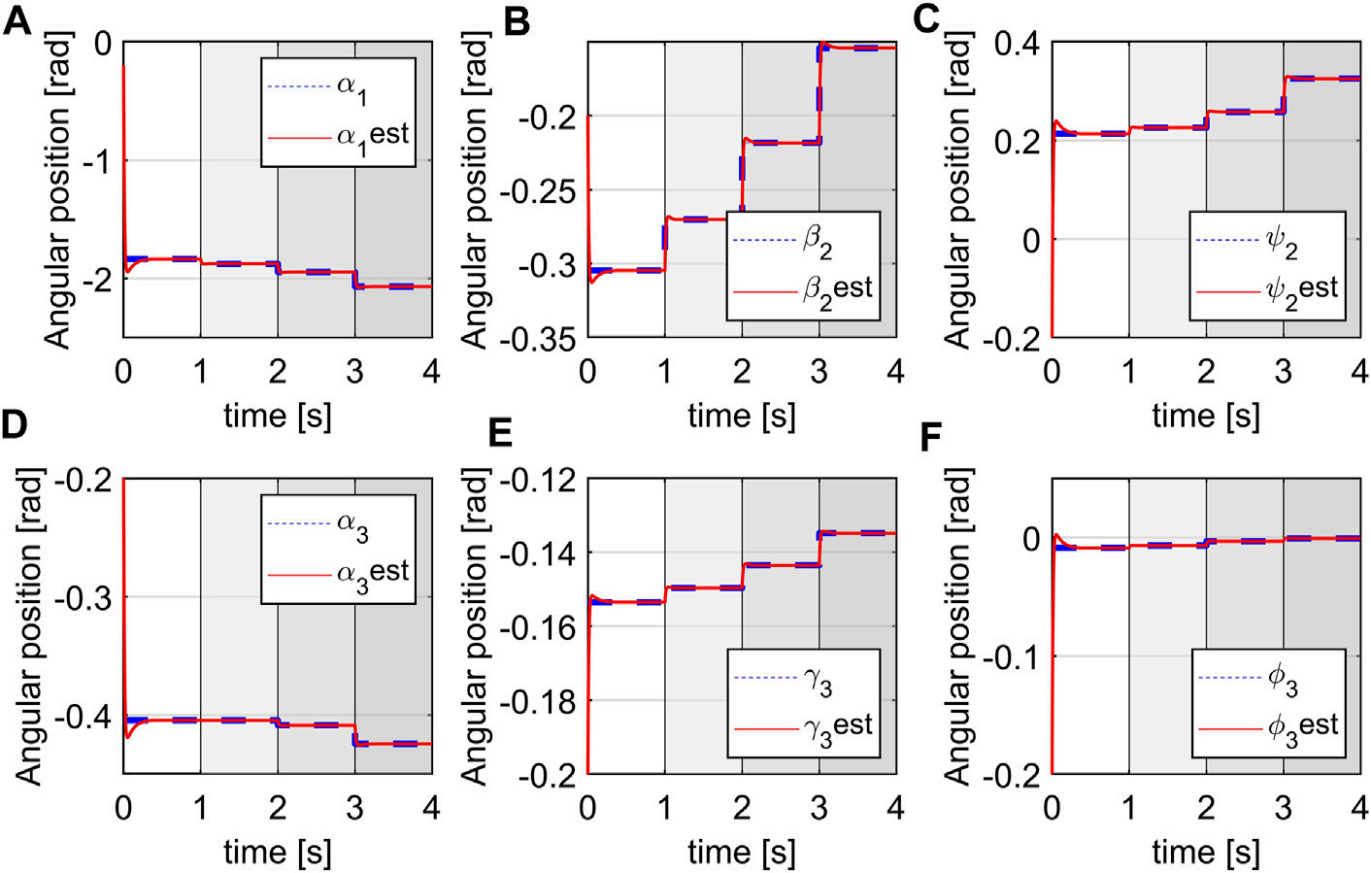
Estimation of space variables, **(A)** *q*_1_, **(B)** *q*_7_, **(C)** *q*_9_, **(D)** *q*_11_, **(E)** *q*_13_, **(F)** *q*_15_.

Simulation 1: The simulation occurred over 4 s and is shown in Figure 10. From the initial time, after each second, the system switched to a new subsystem which is illustrated by a different shading from the light to the darker gray with the darker segments corresponding to a higher pressure. There will be, in total, 15 generalized coordinates to track but due to space considerations, only the estimates of a few representative variables are shown. We can see that from their initial values at −0.2 rad, the estimated variables returned by the observer quickly converge to the true states (from the simulation of the plant), in every subsystem.

**FIGURE 11:**
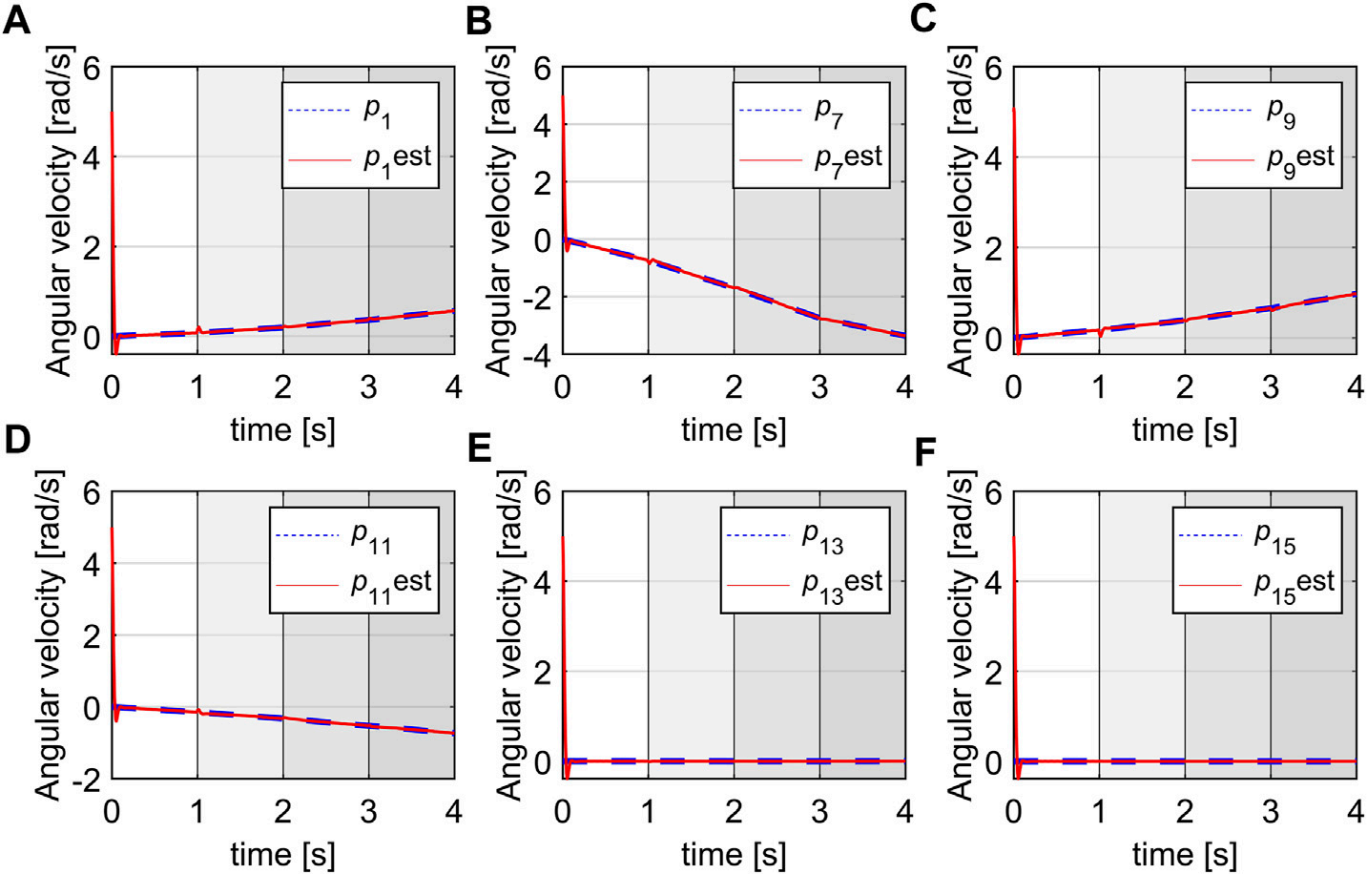
Estimation of the rate of changes of space variables, **(A)** *p*_1_, **(B)** *p*_7_, **(C)** *p*_9_, **(D)** *p*_11_, **(E)** *p*_13_, **(F)** *p*_15_.

Simulation 2: The simulation also ran for 4 s and is shown in Figure 11. It can be observed that the estimates of the rates of changes of the space variables can quickly converge to the true values from their initial values at 5, and without steady-state errors. There are definitely some biases when switching between the subsystems but the steady estimates will be obtained within a short time after entering a new subsystem. Note that the convergence can be smoother at switching points if we linearize the robot by a higher number of subsystems (at more pressure points) but the resulting system will be more complex. In this case, the biases happen in a short time with low magnitudes so their effects are negligible.

**FIGURE 12:**
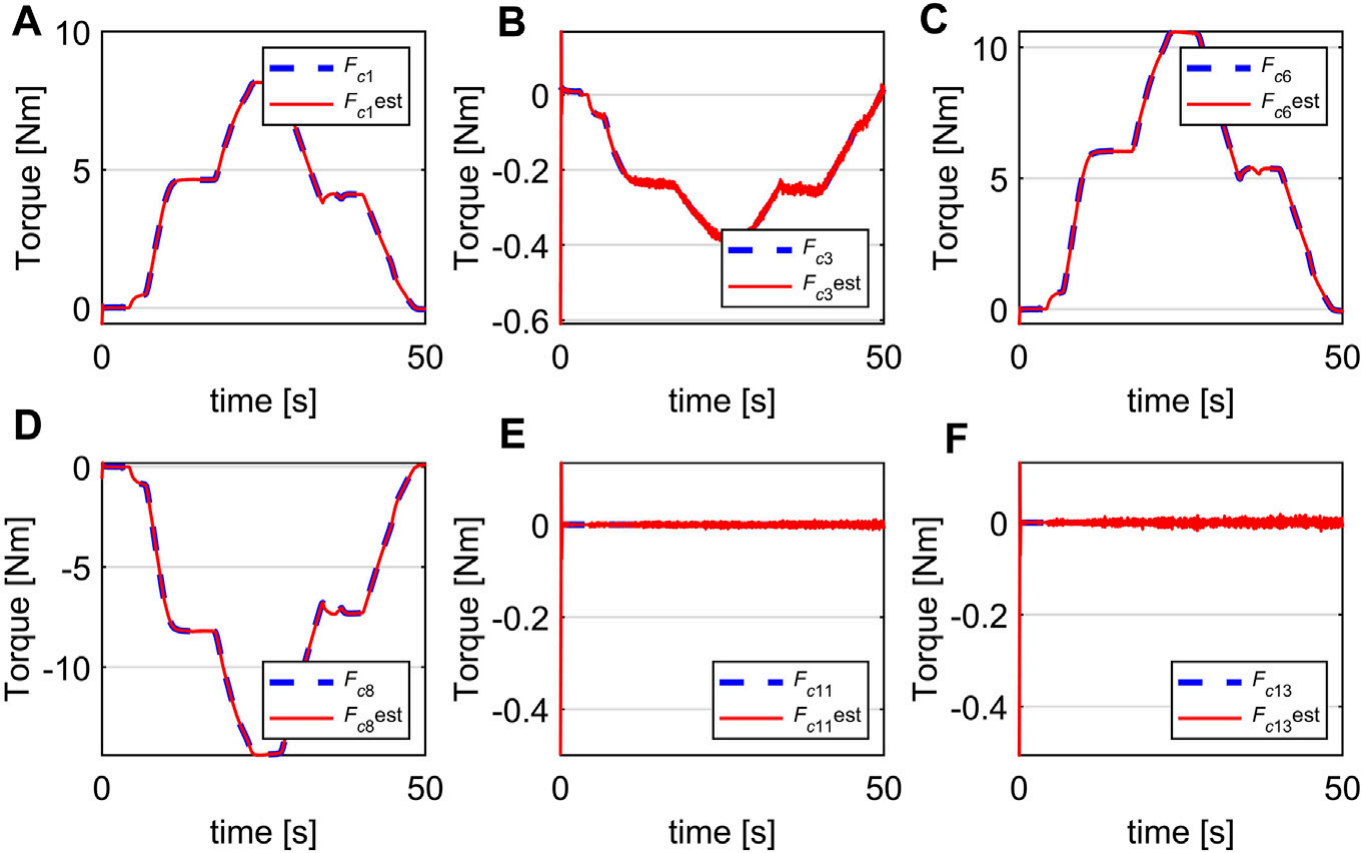
Estimation of generalized contact force, **(A)** *F*_*c*1_, **(B)** *F*_*c*3_, **(C)** *F*_*c*6_, **(D)** *F*_*c*8_, **(E)** *F*_*c*11_, **(F)** *F*_*c*13_.

Experiment 1: The result is shown in Figure 12 where the estimated generalized contact forces are red lines and the ground truth are blue lines. The initial values of estimated contact forces were set at −0.5 Nm, while the initial values of the real generalized contact force were at 0 Nm (there is no contact at the initial time). It can be seen that the observer successfully estimates the generalized contact forces in this experiment with fast convergence. Note that since the contact was at disc 3, the generalized contact forces for all the discs being distal to disc three were zero. Therefore, the real and estimated forces for disc four converge to zero as shown in Figure 12E and Figure 12F. There is some chattering in the estimated forces but due to their low magnitudes, this can be ignored.

**FIGURE 13:**
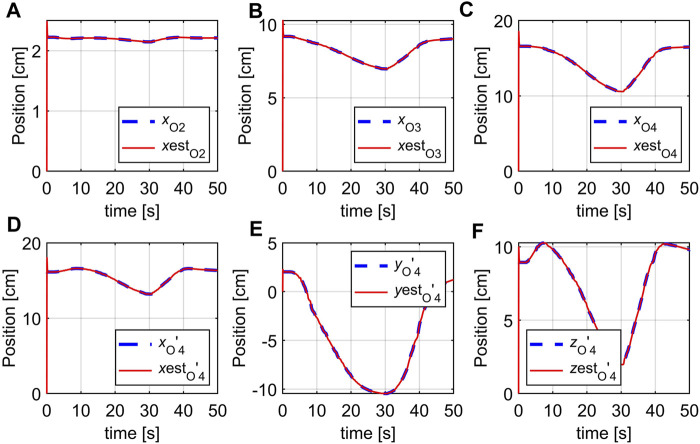
Estimation of marker positions, **(A)** *y*_1_, **(B)** *y*_6_, **(C)** *y*_11_, **(D)** *y*_13_, **(E)** *y*_14_, **(F)** *y*_15_.

Experiment 2: The robot pose is defined as the *X*, *Y*, *Z* coordinates of the seven points that coincide with the markers attached on the body of the robot. The 3D location of the markers are shown as blue lines in Figure 13 while their estimates (provided by the observer) are red lines. To save space, only the estimates of certain selected points ( $O_{2},O_{3},O_{4},O_{4}^{\prime }$ ) are shown in this experiment. We can see that the poses of the soft robot, represented by marker coordinates are well estimated. The estimated coordinates quickly converge to the marker coordinates from their initial values at zero. This indicates that the estimated states of the real robot obtained by the observer are plausible and can be used for model-based control design and implementation.

## 4 Conclusion

This paper introduces an observer method to estimate the generalized states and contact forces for a fabric-reinforced inflatable robot. The proposed observer is a switched linear observer and the switching condition only requires a simple measurement of the chamber pressure. It is based on the disc-thread discretization of the continuum soft robot. An SPV model is developed to equally describe the nonlinear and the hysteretic behavior of the robot. Simulations and experiments are performed to examine the performance of the observer. The results show that the observer works well in simulation by driving the estimated states to the true states in static poses and during movements. The results from the first experiment indicate that the proposed observer can precisely estimate the generalized contact forces during a contact event. The second experimental result shows that by closely estimating the true position of the markers, the observer successfully estimates the system states of the robot. This observer can serve to provide estimates of quantities that are very difficult to measure by sensors. In addition to providing a solution for soft robot perception, by applying this modeling approach and observer design, we may exploit well-developed model-based linear control theory for a class of inflatable soft robots. Although the control schemes for soft robots are not exactly like those for traditional robots, the ability to apply traditional control algorithms can provide stability guarantees and could save considerable effort in controller design. Our future work will develop another sensing approach that can replace the motion capture system and allows the observer to run in real-time.

## Data Availability

The raw data supporting the conclusions of this article will be made available by the authors, without undue reservation.
